# Bionanomaterials or Nanobiomaterials: Differences in Definitions and Applications

**DOI:** 10.3390/jfb16090351

**Published:** 2025-09-18

**Authors:** Bogdan Walkowiak, Małgorzata Siatkowska, Piotr Komorowski

**Affiliations:** Department of Biophysics, Institute of Materials Science and Engineering, Lodz University of Technology, Stefanowskiego 1/15, 90-924 Lodz, Poland; malgorzata.siatkowska@p.lodz.pl (M.S.); piotr.komorowski@p.lodz.pl (P.K.)

**Keywords:** biotechnology, nanotechnology, bionanomaterial, nanobiomaterial

## Abstract

Since the turn of the century, we have witnessed an extremely intensive development of biotechnology and nanotechnology, which, in terms of intensity can only be compared to the development of information technology and the resulting emergence of artificial intelligence. In the present review, we deliberately omit the development of information technology and artificial intelligence. Instead, our interest is focused on bionanomaterials and nanobiomaterials, their production and applications, and, in particular, the different meanings of these terms. We adopted an analysis of the literature published between January 2000 and May 2025, available in PubMed. The database was searched for selected areas: types (origin, structure, and function), manufacturing methods (chemical, physicochemical, and biological), and applications (medicine/pharmacy, textile technology, cosmetology, and agriculture/environment). Our findings revealed a significant increase in the number of publications for both terms, with nanobiomaterials predominating. The authors of the publications included in PubMed clearly outline the separation of meanings of both concepts, despite the lack of normative regulations in this regard. Nanoparticles are the most commonly represented type in the use of both terms, and drug delivery is a dominant application. However, it is worth noting the lack of nanobiomaterials in the agricultural/environmental application categories. Despite the enormous similarity between the terms “nanobiomaterials” and “bionanomaterials,” both in terms of nomenclature and application, there is a significant difference resulting from the manufacturing technologies and applications used. The term “nanobiomaterials” should be assigned only to biomaterials, in accordance with the definition of a biomaterial, regardless of their manufacturing technology, while the term “bionanomaterials” should be applied to all products of bionanotechnology, excluding products used as biomaterials.

## 1. Introduction

**Figure 1:**
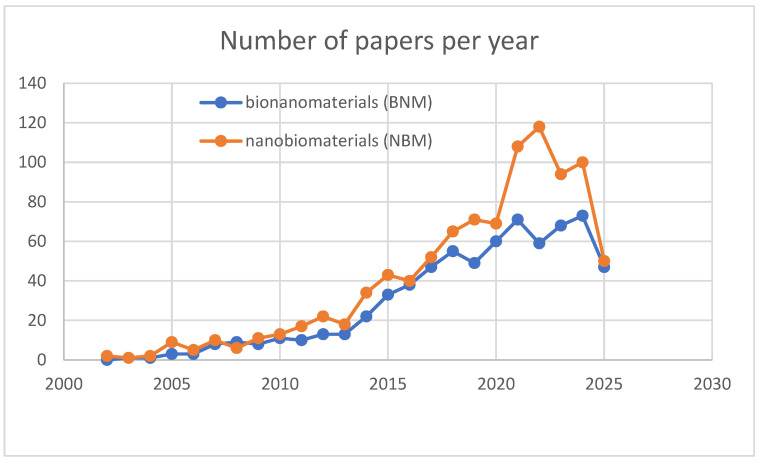
Number of papers per year containing the terms bionanomaterials (BNM) and nanobiomaterials (NBM) found in the PubMed database.

The successes of artificial intelligence resulting from the intensive development of information technology are currently effectively attracting our attention and making a stunning impression. Progress in nanotechnology and biotechnology, which had been in the spotlight since the turn of the century, has been somewhat overshadowed. Although these areas seem very distant from each other, they have a lot in common and interact with each other. The progress of information technology would not be possible without the progress in materials engineering, just as the current dynamic development in the area of nanotechnology and biotechnology could not take place without the use of information technology and artificial intelligence achievements. An example is the ease of access to published information and the speed with which it can be processed. From this very short argument, the importance of mutual influence and stimulation of the development of various technologies results, which, of course translate into economic and social benefits. While these benefits may not always fully address environmental concerns, we believe that economic and social goals can be reconciled with respect for the environment in which we live. Let us leave information technology and artificial intelligence aside and focus on products emerging at the intersection of biotechnology and nanotechnology, i.e., products resulting from the interaction of both technologies. We mean here materials referred to as bionanomaterials and nanobiomaterials. We noticed that there are currently no available normative regulations regarding these concepts. For example, we found no mention of a definition of these terms in the ISO/TS 80004-1:2023 standard (Nanotechnologies—Vocabulary Part 1: Core vocabulary standard) [1]. However, the ISO/TS 80004-5:2011 standard (Nanotechnologies—Vocabulary Part 5: Nano/bio interface standard [2]) mentions the terms bionanotechnology and nanobiotechnology, as well as the terms bio-inspired nanotechnology and biomimetic nanotechnology. Other standards on nanomaterials, including technical committees ISO/TC 229 Nanotechnologies [3], ISO/TS 13329:2024 Nanomaterials [4], or the Standards for Nanotechnology of the National Nanotechnology Initiative [5], also do not include definitions of these terms. Similarly, standards on biotechnology also do not address them either. Due to the lack of formal definitions, we decided to investigate how these concepts are treated by the authors of scientific reports and, on this basis, attempt to differentiate these concepts or assume there are no differences in their meaning. Even a cursory analysis of the PubMed [6] database indicates that the authors of scientific reports tend to treat both concepts separately. We conducted the analysis for the time period from January 2000 to May 2025 (see Figure 1). When asked about bionanomaterials, we received 684 responses, while nanobiomaterials received 939 responses. It is also worth noting that when both terms were searched together, we received only two responses, which suggests that the authors use these terms very selectively.

The following literature review allows us to define the concepts of bionanomaterials and nanobiomaterials, which will also allow us to differentiate them with respect to technological production processes and application areas. Finally, we will try to consider whether the situation could be simplified by adopting a solution in which both concepts are identical, i.e., there is no difference between them.

## 2. Biomaterials

Let us begin our considerations with the concept of biomaterials. Numerous definitions of this term can be cited, ranging from brief to extensive, but for our purposes, let use the very general definition: “Biomaterials are natural or synthetic materials designed to be compatible with the human body and used in a biological environment and are generally used in medical and dental applications” [7]. This definition should be further expanded to include materials used in veterinary medicine [8]. Obviously, this definition does not include the types of biomaterials, their production methods, and applications, as can be read in the following sections.

### 2.1. Types of Biomaterials

Biomaterials can be categorized into four main groups: ceramics, metals, polymers, and composites [9]. Within these groups, we find natural and synthetic materials, as well as those that are highly biocompatible, bioactive, or biodegradable. Ceramic biomaterials are characterized by their high hardness, significant corrosion resistance and good biocompatibility, including, among others: aluminum oxide (Al_2_O_3_) [10], bioactive glass [11], and apatite, including hydroxyapatites [12], as well as carbon materials [13], including carbon layers in the form of diamond-like carbon structures (DLC) [14] or nanocrystalline diamonds (NCD) [15]. Metallic biomaterials are characterized by high mechanical strength and plasticity, including, among others: medical steel, titanium alloys, and cobalt alloys [16,17]. Polymeric biomaterials, that offer a wide range of properties including: polyetheretherketone (PEEK) [16,18], polyethylene (PE) [19], polypropylene (PP) [20], polylactide (PLA) [21], and polytetrafluoroethylene (PTFE) [22]. Composite biomaterials, which are a combination of various materials to obtain the desired properties, include: ceramic-polymer composites [23], metal-polymer composites [24], as well as DLC or NCD carbon coatings [14,15]. The selection of the appropriate biomaterial always depends on the intended function and the application of the manufactured medical device.

### 2.2. Methods of Producing Biomaterials

Biomaterial production is a key step that involves processing the source material to transform it into a high-quality, biocompatible product known as medical-grade biomaterial. The resulting biomaterial is used to produce a medical device that meets the requirements of the ISO 10993 standard [25] and is intended for a specific clinical application. The following methods are used in the production of biomaterials: (a) ceramic forming by uniaxial pressing, extrusion, injection molding, casting, and sintering [26], (b) metalworking by subtractive [27] and additive [28] methods, (c) polymer formation, including solvent casting, molecular leaching, gas foaming, freeze-drying, molecular self-assembly, electrospinning [29], formation of sponges, hydrogels, also for injection (injectable hydrogels) [30], and 3D printing, including bioprinting [29,30], (d) obtaining biopolymers from nature, including the use of animal tissues [31], as well as plant tissues and microorganisms [32], taking into account their appropriate processing, (e) nanofabrication allowing the creation of structures of nanometric dimensions (nanostructures) referred to as bionanomaterials (BNM) [33,34].

Following biomaterial production, the next step involves processing and modification, leading to composite biomaterials with enhanced properties that better meet the clinical requirements for future medical devices. This stage includes, for example: (a) production of biocompatible coatings on the surface of biomaterial by sputtering [35], application of polymer or ceramic coatings [36], carbon layers [14,15], and doped carbon layers [37], (b) combining biomaterials using various techniques to obtain composite structures [38], including biodegradable ones based on magnesium [39].

### 2.3. Applications of Biomaterials

Biomaterials are widely used in human and veterinary medicine, dentistry, and tissue engineering, where they are used to produce implants, prostheses, surgical sutures, dressings, and bone regeneration materials. They are used to replace damaged tissues and organs and to support healing and regeneration processes. Examples of biomaterial applications in medicine include: (a) dental [40], orthopedic [41], neurological [42], spine surgery [43], peripheral nerve regeneration [44], ophthalmic [45], vascular [46], and cardiac surgery [47] implants; (b) surgical sutures [48], meshes [49] and membranes [50], and materials for the reconstruction of bone defects [51]; (c) dressings, also in the form of hydrogels, for the treatment of wounds, including burns, pressure sores, ulcers [52], and diabetes [53]; (d) scaffolds for cells in tissue regeneration [54] and biomaterials for medical imaging [55].

## 3. Nanomaterials

In accordance with the recommendation of the European Commission on 11 June 2022 [56]: “‘Nanomaterial’ means a natural, incidental or manufactured material consisting of solid particles that are present, either on their own or as identifiable constituent particles in aggregates or agglomerates, and where 50% or more of these particles in the number-based size distribution fulfill at least one of the following conditions: (a) one or more external dimensions of the particle are in the size range 1 nm to 100 nm; (b) the particle has an elongated shape, such as a rod, fiber or tube, where two external dimensions are smaller than 1 nm and the other dimension is larger than 100 nm; (c) the particle has a plate-like shape, where one external dimension is smaller than 1 nm and the other dimensions are larger than 100 nm.” Detailed guidance on the implementation of this commission recommendation is available in the Joint Research Center (JRC) Science for Policy report [57]. A characteristic feature of nanoparticles is a large surface-to-volume ratio, which results in a high percentage of atoms present on the surface [58], up to the limiting conditions when all atoms are available on the surface (e.g., graphene) [59]. The second characteristic feature is the ubiquity of quantum effects controlling interactions between atoms and molecules on the nanometric scale [60]. Both of these features are responsible for the unique physicochemical properties of nanomaterials, which are completely different from the physicochemical properties of their macroscopic counterparts.

### 3.1. Types of Nanomaterials

Nanomaterials can be classified in several ways—depending on their shape, structure, chemical composition, or origin. Based on the number of dimensions, they can be divided into: (a) nanoparticles (e.g., quantum dots, dendrimers, and liposomes) with all dimensions ≤ 100 nm (0 D), (b) nanofibers and nanorods with two dimensions ≤ 100 nm (1D), (c) nanolayers with one dimension ≤ 100 nm (2D), and (d) three-dimensional nanostructures composed of nanoelements (3D) [61,62]. In terms of chemical composition, nanomaterials are usually divided into carbon, metal and metal oxide, ceramic, polymer, and composite [62,63]. Depending on their origin, nanomaterials can be classified as natural (volcanic eruptions and weathering of minerals), synthetic (intentionally produced—engineered), and incidental (created accidentally, e.g., in combustion or detonation processes) [61,63].

### 3.2. Methods of Producing Nanomaterials

The production of nanomaterials is carried out according to one of two strategies: “top-down” or “bottom-up” methods. The “top-down” strategy, which involves the fragmentation of macroscopic material, includes ball milling, thermal decomposition, lithography, laser ablation, and etching. The “bottom-up” strategy, which involves building nanostructures from atoms or molecules, involves chemical synthesis methods, deposition methods, including Chemical Vapor Deposition (CVD), Physical Vapor Deposition (PVD), Plasma Enhanced Chemical Vapor Deposition (PECVD) and their modifications, sol–gel methods, self-assembly methods, electrodeposition, synthesis using microorganisms, and green synthesis methods [61,62,63].

### 3.3. Applications of Nanomaterials

Nanomaterials have a wide range of applications across various fields due to their unique physicochemical properties and biological effects. In medicine and pharmacy, nanomaterials are used for drug delivery [64,65], imaging diagnostics [65], tissue engineering [66], and the production of antimicrobial coatings [67]. Examples of applications in electronics and computer science include nanoelectronics, including electronic components such as transistors [68], new types of memory [69], sensors [70], screens, and displays [71]. In the energy sector, nanomaterials are used to produce photovoltaic cells [72] and supercapacitors [73], while in chemistry, they serve as efficient and selective catalysts [74]. The industrial applications of nanomaterials are very wide, especially in the automotive industry [75], and also through their use in new building materials [76], nanocomposites [77], protective coatings [78], functional textiles [79], as well as active packaging [80] and food quality sensors [81], in cosmetic products [82], and household chemicals [83]. Due to the widespread use of nanomaterials, their potential adverse impact on the environment and our health should be taken into account, and this potential threat must be constantly monitored [84].

## 4. Nanobiomaterials and Bionanomaterials

### 4.1. Nanobiomaterials

Against the backdrop of rapidly developing nanotechnology, the need to implement this technology for the production of biomaterials has inevitably arisen. The first available information using the term “nanobiomaterial,” in the form of review papers available only in Chinese, appeared in 2002 and concerned the nanostructuring of scaffolds for tissue culture [85,86]. Due to the review format of the publication, it should be assumed that original research papers on the production and use of nanobiomaterials, although not necessarily named as such, appeared several years earlier, which clearly confirms the turn of the century as the emergence of nanobiomaterials. Nanoparticles are characterized by dimensions similar to those of bioparticles (e.g., proteins and nucleic acids) and cellular structures (e.g., membranes and cytoskeletal structures). The integration of nanoparticles with unique electronic, photonic, and catalytic properties with biomaterials with unique recognition, catalytic, and inhibitory properties leads to the creation of new hybrid nanobiomaterials with synergistic properties and functions [87]. In this sense, nanobiomaterials should be considered a product of intentionally combined efforts in the fields of nanotechnology and biomaterials engineering, which has created enormous opportunities for improving the prevention, diagnosis, and treatment of various diseases. Nanobiomaterials represent a new class of extraordinary biomaterials with unique structures and properties currently finding widespread biomedical applications [88]. Examples of nanobiomaterial types, their structures and functions, as well as methods of fabrication and application are summarized in Table 1 for easier comparison with bionanomaterials.

### 4.2. Bionanomaterials

As a result of the intensive development of biotechnology and the unique properties of its products, the possibility of combining nanotechnology and biotechnology, known as nanobiotechnology, has clearly emerged. This has reduced the harmful ecological impact of chemical synthesis of nanomaterials [89] and resulted in the development of a new class of nanomaterials known as bionanomaterials [90]. Bionanomaterials are materials composed almost entirely or largely of biological molecules produced by cells, such as peptides and proteins, nucleic acids, lipids, oligosaccharides, as well as secondary metabolites and viruses, forming molecular structures with nanometric dimensions [91]. This class also includes metal and metal oxide nanoparticles synthesized by microorganisms and plants [92]. Bionanomaterials possess unique structural, chemical, physical, optical, functional, mechanical, and electrical properties that distinguish them from macroscopic materials due to their exceptionally small size. These properties are valuable in various medical applications, but bionanomaterials are also widely used in other sectors of the economy [93]. A list of examples of types of bionanomaterials, their structures and functions, as well as methods of production and application, is collected in Table 1 for easier comparison with nanobiomaterials.

**Table 1:** Summary of selected exemplary information on nanobiomaterials and bionanomaterials.

Features of NBM and BNM			Nanobiomaterials	Bionanomaterials
			Selected Examples	
types	origin	natural	bacterial extracellular vesicles as natural nanomaterials in disease diagnosis and therapeutics [94], microbial cellulose for healing of wounds [95],	bio-based nanomaterials [91], natural biopolymers [96],
		synthetic	hydroxyapatite (HA) Al_2_O_3_–TiO_2_ [97], synthetic polymer-based nanocomposites [98],	synthetic biopolymers [96], synthetic polymer-based nanocomposites [98]
		hybrid	hybrid nano hydrogel for bone regeneration [99], peroxidase-mimic GSF@AuNPs hybrid nanoparticles [100],	protein-based functional hybrid bionanomaterials [101], hybrid chitosan-cerium oxide nanoparticles [102],
	structure	nanoparticles	TiO_2_ nanoparticles—a promising candidate for wound healing applications [103], collagen-I@AuNPs for treating skin injuries [104],	bioinspired nanoparticles emerge for medical use [105], protein corona formation on single nanoparticles for theranostic applications [106],
		nanofibers/nanowires	MnO_2_ nanofibers for selective and sensitive detection of biomolecules [107], nanowires for selective detection of chloramphenicol [108],	cellulose nanofibers and sodium alginate composite with antibacterial properties [109], ATP as building blocks for the self-assembly of excitonic nanowires [110],
		nanocoatings	nanocoatings and their composites in dentistry [111], ZnO based nano-architectures, films and coatings for biomedical applications [112],	gentamicin loaded multilayers modified titanium coatings for prevention of implant infection [113], polysaccharide based coatings for fruit preservation [114],
		nanohydrogels	halofuginone–silver thermosensitive nanohydrogels with antibacterial and anti-inflammatory properties [115], nanogels as promising nanosystems to treat a wide range of acute and chronic healthcare scenarios [116],	chitosan-inspired (nano)hydrogels [117] nanogels as carriers for medical applications [118],
	functions	biocompatible	biocompatibility of nanobiomaterials [119], biocompatibility of chitosan-carbon nanocage hybrids for sustained drug release [120],	conjugation of nanoparticles with the biological molecules makes them more biocompatible [91], biocompatible bionanomaterials for food packing [121],
		bioactive	highly bioactive nanoparticle for activating osteogenesis [122], osteogenic nanometric bioactive glass particles [123],	advanced bioactive nanomaterials for diagnosis and treatment [124], bioactive polymers and nanobiomaterials composites [125],
		biodegradable	bacterial cellulose—nanobiomaterial for biodegradable face masks [126], biodegradable PLA nanoplatforms as coatings for cardiovascular stents [127],	biodegradable poly(amino acid)-gold-magnetic complex for photothermal treatment [128], biodegradable PEG-dendritic block copolymers [129],
		targeted	siRNA drug delivery strategies [130], extracellular vesicles in targeted delivery towards specific cells [131],	phage particles for targeted delivery of personalized neoantigen vaccines [132], molecularly targeted viral nanoparticles as tools for imaging cancer [133],
manufacturing methods	chemical	polymerization	on-surface polymerization in living cells [134], techniques for chemical and biological synthesis of polymeric nanoparticles [135],	techniques for chemical and biological synthesis of polymeric nanoparticles [135], microwave-assisted click polymerization of cyclic oligomers [136],
		functionalization	surface functionalization of nanobiomaterials for tissue engineering and regenerative medicine [137], functionalization of tissue-specific bioinks [138],	functionalization of green synthesized bionanomaterials [139], lignocellulosic bionanomaterials for biosensor applications [140],
	physicochemical	radiation	osteoconductive effect of a nanocomposite membrane treated with UV radiation [141], designing nanostructured Ti6Al4V with directed irradiation synthesis [142],	irradiation effects in polymer composites for their conversion into hybrids [143], surface modification of polymers by exposure to extreme ultraviolet radiation [144],
		electrospinning	polyvinyl alcohol/chitosan nanofibrous films by electrospinning method [145], application of electrospinning technology in the biomedical field [146],	electrospinning technology, machine learning, and control approaches [147],
	chemical/biological synthesis	synthesis/green synthesis	synthesis of copper nanobiomaterials [148], synthesis of biomimetic nano/submicro-fibrous tubes for potential small-diameter vascular grafts [149],	green synthesis of hydrogel scaffolds [150], green synthesis with root extract [151], self-assembling bionanomaterials [152],
applications	medicine/pharmacy and textile technology	drug delivery	nanobiomaterials in spatio-temporal controlled drug delivery for lungs [153], rotavirus as a vector for heterologous peptides, drug delivery, and production of nanobiomaterials [154],	drug delivery with supramolecular amphiphilic macrocycle nanoparticles [155], plant virus nanoparticles as nanocarriers for drug delivery and imaging [156],
		imaging	fluorescence bioimaging of molecular fluorophores [157], hyperspectral imaging for label free detection of nanobiomaterials [158],	bionanomaterials and systems for enhanced bioimaging in biomedical applications [159], viral nanoparticles for in vivo tumor imaging [160],
		scaffolds/tissue engineering	3D bioprinting and nanotechnology in tissue engineering scaffolds [161], surface modification by nanobiomaterials for vascular tissue engineering [162],	extending the versatility of bionanomaterial scaffolds [163], amyloid fibrils as a nanoscaffold for enzyme immobilization [164],
		dressings	polyvinyl alcohol/chitosan nanofibrous films by electrospinning method for wound dressings [144], nanobiomaterials for vascular biology and wound management [165],	bionanomaterials for skin regeneration [166], bionanomaterials in wound dressings [167],
		diagnostics	nanobiomaterials for point-of-care diagnostics [168], diagnostics strategies based on engineered nanobiomaterials [169],	clinical in vivo nanodiagnostics [170], bionanomaterials for diagnosis and therapy of SARS-CoV-2 [171],
		textile/fabrics	composites based on CNTs and 2D material coated fabric sensors [172], silver plating on polyester and cotton blended fabric [173],	biomimicry in the field of textile technology [174],
	cosmetology	cosmetics	dermal delivery of drugs and cosmetics [175], nanobiomaterials in cosmetics [176],	green synthesized nanomaterials for cosmetics [177], liposomes in cosmetics [178],
	agriculture and the environment	pesticides		bionanomaterials towards the environmental and agricultural domain [179], nanopesticide application in crop protection [180],
		remediation		recent advances in nanoremediation [181], remediation of microplastics using bionanomaterials [182],
		packaging	application of an organic silver-metal framework modified with sodium alginate in packaging [183], nanobiomaterials for food packaging sensor applications [184],	bionanomaterials for development of sustainable food packaging [157], (bio)nanotechnology in food science [185],

### 4.3. Discussion

The concepts of nanobiomaterials and bionanomaterials derive from technological advances in nanotechnology and biotechnology and the specific demands of biomaterials engineering. Nanotechnology arose from the scientific achievements of the 20th century in physics and chemistry. At the turn of the 20th and 21st centuries, nanotechnology matured to allow the use of biotechnological solutions, and thus products known as bionanomaterials emerged. A characteristic feature of bionanomaterials is the use of biotechnological methods for their production and the possession of properties typical of nanomaterials. Unlike bionanomaterials, nanobiomaterials are the products of biomaterials engineering, which utilize advances in biotechnology and nanotechnology to obtain qualitatively new biomaterials intended for biomedical applications. In this case, the specific biomedical application is decisive, as opposed to the broadly understood possibilities of bionanomaterials.

When analyzing the data presented in Table 1, it becomes evident that in most cases the information regarding nanobiomaterials and bionanomaterials is either similar or identical. The conclusion may arise that distinguishing these terms is not particularly significant. In some cases, this likely results from a lack of consequences in the use of these terms, and sometimes they are used inconsistently with their actual meaning. For example, the paper [181] discusses the use of nanotechnology and bionanotechnology products for soil remediation. In the light of the above considerations, bionanotechnology products should be referred to as bionanomaterials, while the authors use the term nanobiomaterials in their keywords. In another work [186], both terms are used interchangeably. The paper indicates that the authors are interested in biotechnologically modified 2D nanosheets for a wide range of industrial applications, and the correct term for these products would be bionanomaterials. The opposite situation can be found in another work [187], where the technology of producing nanomaterials for biomedical applications, i.e., a nanobiomaterial, is presented and such a term is included among the keywords, while the title of the publication contains the term bionanomaterials.

A noticeable difference appears when the use of the term “nanobiomaterials”, as well as “biomaterials”, does not make much sense, while the meaning of “nanomaterials”, and, in particular, “bionanomaterials”, are crucial. For example, in relation to plant protection products and soil remediation, we did not find any examples of nanobiomaterials used in these application areas, while the term “bionanomaterials” was used in this context. It is worth noting the possible false-positive responses provided by PubMed in this case. For the queries “bionanomaterials” and “pesticides,” some results were returned due to the presence of the term “bionanomaterials” in the authors’ affiliation (Bionanomaterials and Bioengineering Group) [188] or the appearance of this term in the reference list [146].

**Figure 2:**
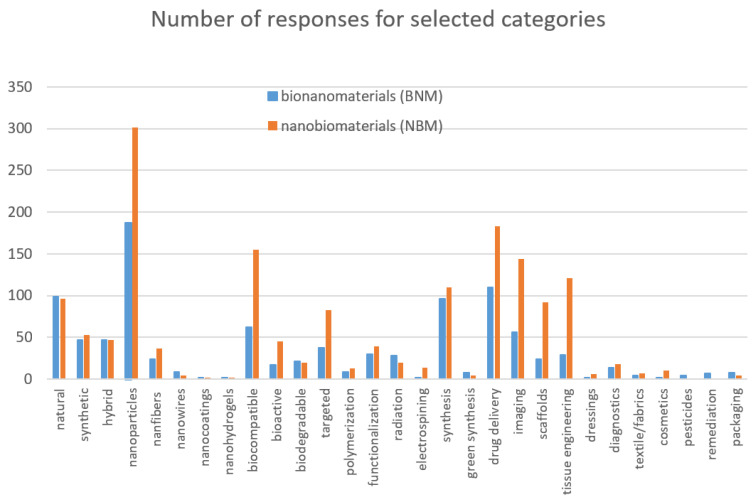
The number of the PubMed database responses for selected categories corresponding to the categories in Table 1.

To facilitate tracking the frequency of occurrence of the terms bionanomaterials or nanobiomaterials in the PubMed database in combination with the categories contained in Table 1, a relevant summary is provided below in graphical form (Figure 2).

From this graph, it is clear that the most frequently represented type of both bionanomaterials and nanobiomaterials is in the form of nanoparticles, while the most frequently cited application is drug delivery, with biomedical applications clearly predominating. Some categories are very poorly represented for both terms. It can also be concluded that although nanobiomaterials dominate, all categories are represented similarly for both terms.

Of course, it can be concluded that the nomenclature issue is not significant, especially since we encounter similar dilemmas when using the terms “particle” and “molecule.” The concept of particles is reserved for objects of physics, such as elementary particles or tiny material objects. The concept of molecules has found its place in chemistry and describes multiatomic objects. This division would not pose any problems were it not for the emergence of nanotechnology. Nanotechnology deals with objects much larger than elementary particles but often comparable to many chemical molecules, especially when they occur in the form of polymers, including biopolymers. This situation often leads to confusion, as biopolymers are sometimes treated as chemical molecules, particularly in biochemistry, and at other times as nanoparticles resulting from applications of bionanotechnology.

However, the problem we mentioned with the nomenclature of nanobiomaterials and bionanomaterials has a slightly different meaning. It concerns the same field of technology, namely nanotechnology. Here, precision in the use of these terms would be required. Therefore, we propose that products of nanotechnology or bionanotechnology used in production and applications as biomaterials, in accordance with the definition of biomaterial, should be referred to as nanobiomaterials. In contrast, bionanotechnology products resulting from the application of biotechnology, or even inspired by the functioning of biological objects, should be referred to as bionanomaterials. This distinction will help avoid many unnecessary misunderstandings in the future and improve clarity within the field.

## 5. Conclusions

Despite the enormous similarity between the terms “nanobiomaterials” and “bionanomaterials,” both in terms of nomenclature and application, there is a significant difference that arises from the manufacturing technologies and applications used. We propose that the term “nanobiomaterials” be assigned exclusively to biomaterials, in accordance with the definition of a biomaterial, regardless of their manufacturing technology, while the term “bionanomaterials” should be applied to all products of bionanotechnology, excluding those used as biomaterials. It should be acknowledged that these terms are not identical and are not interchangeable. We believe it is essential for the relevant standardization bodies to take a position on the issue raised in this review and provide clear and precise definitions for both terms.

## Data Availability

No new data was created or analyzed in this study. Data sharing is not applicable to this article.
